# The relationship between ostracism and negative risk-taking behavior: the role of ego depletion and physical exercise

**DOI:** 10.3389/fpsyg.2024.1332351

**Published:** 2024-01-24

**Authors:** Fang Chen, Jinhong Wang, Heng Gao, Yadi Zeng, Ziwei Li, Hongyu Zou

**Affiliations:** ^1^College of Education and Sports Sciences, Yangtze University, Jingzhou, China; ^2^School of Psychology, South China Normal University, Guangzhou, China; ^3^School of Psychology, Shaanxi Normal University, Xi’an, China

**Keywords:** college students, ostracism, ego depletion, physical exercise, negative risk-taking behavior

## Abstract

**Background:**

As a major public health problem globally, negative risk-taking behavior of college students may be related to their ostracism experience, but the reason for this association is unclear. Based on the limited resource theory, combined with the integrative model of athletic performance, we tested a moderated mediation model in which ego depletion mediated the association between ostracism and risk-taking, and physical exercise moderated the mediation process to examine the mechanisms underlying the association between ostracism and negative risk-taking behavior.

**Methods:**

One thousand three hundred seven students (43% female) from four universities in China were recruited using cluster random sampling. The experience of being ostracized, ego depletion, physical exercise level, and negative risk-taking behavior were measured through an anonymous online questionnaire in “www.sojump.com.”

**Results:**

After controlling for gender and grade in college, ostracism was positively related to negative risk-taking behavior; ego depletion mediated this relationship; and physical exercise level attenuated these direct and indirect relationships.

**Conclusion:**

The results highlight individual risk and protective factors associated with negative risk-taking behavior, and provide new perspectives on ways to prevent and reduce college students’ negative risk-taking behavior.

## 1 Introduction

Negative risk-taking behavior is also often referred to as problematic behavior, dangerous behavior, etc., and mainly include dangerous driving, smoking, drunkenness, truancy, cheating, risky sexy behaviors, aggression, drug abuse, etc. (Hansen and Breivik, 2001). It had become a worldwide major public health problem, and was one of the leading causes for illness and death among people aged 18–24 years (Taghvaee and Mazandarani, 2022). Adolescence, defined as a period of high risk-taking behavior, which generally refers to the ages of 10–24 (Smetana et al., 2006; Sawyer et al., 2018; Zou et al., 2023). Late-adolescent (18–24) college students are at a crucial stage of transition from childhood to early adulthood. This is also a time when risky behaviors, such increasing alcohol use and risky sexual activity, are more common and prevalent (Duell et al., 2018; Liang et al., 2022). They are less mature than adults in terms of life experience, danger recognition and assessment skills, and self-control (Liang et al., 2022; Qiu et al., 2022). These characteristics may encourage college students to engage in negative risk-taking behavior. Therefore, it is important to focus research on this population.

In China, research found that the prevalence of dangerous behaviors, including unhealthy eating behaviors, daily risky behaviors, substance abuse behaviors, risky sexual behaviors among college students overall was 16.85% (Hu et al., 2023). Besides, the prevalence of cheating on exams is 10.6 and 41.1% for coursework and essay writing (Stiles et al., 2017; Wang and Xu, 2021) and is becoming more prevalent with the development of artificial intelligence (e.g., ChatGPT) (Hung and Chen, 2023). This places individuals at risk for physical and mental disease, untimely or needless death, and social issues including poverty, criminality, and academic failure (Taylor and Stupica, 2015; Dou et al., 2020a). It also puts society’s safety at risk (Wagemaker et al., 2020). In view of the current situation and the serious consequences of negative risk-taking behavior, this study aims to explore the susceptibility factors as well as the underlying mechanisms associated with negative risk-taking behaviors among college students.

Recent researchers have explored the causes of risk-taking behavior among college students in terms of personality, environment, and cognition (Jia et al., 2021; Oduaran and Agberotimi, 2021; Tyler and Brownridge, 2022). Among them, peer interactions and relationships are important influences on college students’ negative risk-taking behavior (Chinopfukutwa and Hektner, 2021). Humans are innately social and have a strong need to belong to groups, and to establish relatively positive and stable lasting social relationships with others to keep in physical and mental health (Leary and Baumeister, 1995). According to Niu et al. (2023), college students who are removed from the group are prone to have health and adjustment issues as well as the development of harmful behaviors. The feeling of exclusion is a risk factor for negative risk-taking behavior among college students, according to research that has focused on the relationship between ostracism and risk-taking behavior (Svetieva et al., 2015). However, the mechanisms between this process are still unclear. Therefore, in this study aimed to test the relationship between ostracism and negative risk-taking behavior and its underlying mechanisms in a sample of Chinese university students. This study enriches the related research on ostracism on negative risk-taking behavior of college students, and also provides a feasible direction for the prevention and reduction of negative risk-taking behavior of college students.

### 1.1 Ostracism and negative risk-taking behavior

Ostracism (being excluded and ignored), also known as social exclusion, is a pervasive phenomenon in our daily lives, often resulting in negative emotions and problematic behaviors (Williams, 2009). Ostracized adolescents have been shown to exhibit a higher tendency toward antisocial behavior, risky decision-making, and negative risk-taking behavior, whether the study used questionnaires or experimental virtual contextual methods (Duclos et al., 2013; Xu et al., 2023). The temporal need-threat model suggests that ostracism threatens the fulfillment of basic psychological needs (belonging, self-esteem, control and meaningful existence/need for recognition) (Williams, 2009). It is also a painful emotion and as with most responses to pain, negative emotions increase, including anxiety, sadness, and anger, while positive emotions decrease (Williams, 2007). When individuals are excluded, they feel alienated, helpless, and self-denial, causing dysfunctional cognitive-emotional abilities, which manifests more problem behaviors (Reiter-Scheidl et al., 2018; Zhang et al., 2022). A study on Chinese college students showed that experiencing ostracism depletes college students’ self-esteem. College students with low self-esteem are prone to ignore the risks behind the benefits and thus show more risk-taking behaviors (Meng, 2020).

Buelow and Wirth (2017) conducted two experimental studies with Australian university students. Experiment 1 manipulated exclusion using a network circle paradigm followed by completion of a balloon simulation risk task to measure risk-taking behavior. In Experiment 2, gambling risk-taking behavior was followed by controlled exclusion utilizing Atimia’s exclusion task, which produces exclusion effects by manipulating virtual players to exclude or include participants with word-associated objects. Both experiments’ results demonstrated that respondents who felt excluded exhibited significantly more risk-taking behavior; the impact was larger in Experiment 2, perhaps as a result of the experiment’s more demanding tasks requiring cognitive and self-control (Buelow and Wirth, 2017). The limited resource theory (Baumeister, 2003) provides an ideal framework for this result. The theory posits that human control resources are limited, and a series of maladaptive manifestations may occur by consuming too much self-control resources. Since ostracism, as a negative life event, exposes individuals to stressful and threatening situations that require them to use strategies such as cognitive regulation or emotional control to cope with them, it depletes self-control resources and leads to a failure of self-control, the adoption of unconventional thinking or behavioral activities, and the engagement in negative risk-taking behavior (Muraven et al., 1999). Certainly, ostracism does not always lead to negative experiences and outcomes. For instance, interpersonal alienation due to a higher tendency to be alone or a lack of social interest does not necessarily have a significant correlation with internalizing, externalizing problem behaviors (Coplan et al., 2013). At this point, it is important to explore other potential determinants at this time, such as antecedent anxiety and depression symptoms. In the present study, ostracism relates more to being passive ignored and rejected passively and exists primarily as an external stressors and threatening stimulus. According to the generalized strain theory, the stress experienced by an individual is the main cause of undesirable behavior (Agnew et al., 2002). We thus hypothesize that among college students that ostracism may be positively associated with more negative risk-taking behaviors.

### 1.2 Ego depletion as a mediator

Self-control is a key individual factor influencing adolescents’ negative risk-taking behavior (Jia et al., 2021). However, it is a limited resource, and low reserves create a state known as ego depletion (Tangney et al., 2004; Baumeister and Tice, 2007). Specifically, ego depletion refers to the state of temporary decline in self-control ability or willingness to perform volitional behaviors due to previous self-control tasks or volitional exercises depleting an individual’s limited self-control resources (Bratslavsky et al., 1998; Baumeister and Vohs, 2016). Based on the limited resource theory (Baumeister, 2003), the threats to an individual’s resources or needs from exclusion constitute an external stressors that depletes limited self-control resources and enters ego depletion (Wang and Zhang, 2020). This state is often accompanied by a decrease or failure in self-control, resulting in a range of problematic behaviors (Hagger et al., 2010; Mead et al., 2010). Therefore, ego depletion may mediate the relationship between ostracism and negative risk-taking behavior.

Many scenarios and behaviors produce ego depletion, such as stress coping, negative experiences, emotion regulation, thought suppression, and impulse control (Baumeister et al., 2007). Researches have proven that ostracism appears to be correlated with ego depletion, when individuals perceive ostracism, they need to consume significant self-control resources to regulate the negative thoughts and emotions associated with it, such as stress, sadness, and the desire for revenge (Lee et al., 2016; Kavakli, 2021). Baumeister et al.’s (2005) experiment, with 30 undergraduate students as subjects, manipulated social exclusion by randomly assigning people to receive bogus feedback about the future trajectory of their social lives. Participants in the exclusion group performed worse than those in the acceptance group in subsequent binaural split-listening tasks requiring self-control resources, and that individuals who experience ostracism were prone to develop ego depletion (Baumeister et al., 2005). According to recent research, college students who were socially ostracized and given challenging arithmetic assignments as separate ego depletion tests showed higher ego depletion effects when performing a later self-regulation challenge, which resulted in lower math academic performance (Saldivar, 2018). Thus, individuals deplete resources in social exclusion situations, and thus show relatively low performance on subsequent tasks is an outward manifestation of ego depletion.

Moreover, ego depletion theory suggests that individuals under ego depletion experience a weakening or loss of self-regulation, which increases the likelihood of negative psychological and problematic behaviors (e.g., excessive procrastination, substance dependence, excessive alcohol consumption, violent behaviors, irrational spending, risky sex, and unhealthy dietary habits) (Muraven, 2012; Baumeister, 2014). Research has found that ego depletion, in particular, promotes risk-taking behaviors with immediate rewards and delayed consequences, allowing individuals to prefer immediate gratification over long-term gains (Unger and Stahlberg, 2011). For example, ego depletion increases the probability of online risk-taking and unethical behaviors such as aggressive speech and violent behavior among college students (Ding et al., 2020a). Ego depletion may lead to increased alcohol consumption, failure to quit smoking, unhealthy eating, and risky sexual behavior among college students (Gissubel et al., 2018). In study with adolescents, individuals in ego depletion show higher negative emotions, lower information processing ability, and higher aggression (Chen et al., 2023). Therefore, we assume that ego depletion may mediate the relationship between ostracism and negative risk-taking behavior.

### 1.3 Physical exercise as a moderator

With the development of sport psychology, the protective effects of physical activity on an individual’s physical and mental health are becoming more widely acknowledged. Researchers have documented the role of physical exercise in reducing stress, improving emotional state, promoting cognitive function, and reducing the occurrence and development of psychological and behavioral problems (Jiang et al., 2018; Elbe et al., 2019; Smith and Merwin, 2021). Previous evidence suggests that physical exercise released college students’ stress and was beneficial in reducing internalizing problems such as anxiety and depression, as well as externalizing problems such as risk-taking and impulsivity (Herbert et al., 2020). Meanwhile, physical exercise also has positive effects in reducing withdrawal symptoms, decreasing the desire to smoke, controlling drinking behavior, and reducing antisocial behavior (Abrantes et al., 2017; Jackson and Beaver, 2018; Bock et al., 2019). Similarly, physical exercise was found to be effective in suppressing aggressive behaviors during the COVID-19 in China (Jiang, 2022). The integrative model of athletic performance emphasizes that playing sports produces positive cognitive, emotional, and physiological experiences, improves self-control, and alleviates negative emotions that protect physical and mental health (Huang et al., 2023). This can be seen as a moderating mechanism for physical exercise.

According to the limited resources theory, all self-control activities are dependent on limited resources and that ego depletion occurs when the performance of self-control is impaired (Heatherton and Baumeister, 1996). However, ego depletion is only temporary and energy resources can be recovered (Muraven and Baumeister, 2000). Physical exercise can recover energy resources. Exercise can train individuals’ strength (baseline ability) and endurance (ability to tolerate exertion) to improve self-control and reduce energy loss (Muraven et al., 1999). Previous research has demonstrated that mental energy resources are not only restored but enhanced through sustained exercise behaviors (Finley and Schmeichel, 2019). These energy resources help to broaden the mind and maintain focus, accompanied by more positive emotional experience, which counteracts negative emotions triggered by adverse environmental factors (e.g., ostracism), reduces psychological stress, and alleviate ego depletion (Huang et al., 2023). Additionally, evidence suggests that physical exercise can mitigate the negative effects of external risk environments (e.g., stress, family conflict, school bullying, etc.) on individuals’ mood and cognition (negative emotions, cognitive maladjustments), acting as a stress buffer (Sigfusdottir et al., 2011; Brown and Lawton, 2015; Kirklewski et al., 2020) and further reduces the likelihood of risk-taking behavior among college students (Newman et al., 2017). Therefore, we assume that physical exercise may moderate the relationship between and negative risk-taking behavior, as well as the impact of ego depletion as a mediator of this effect.

### 1.4 The present study

**Figure 1:**
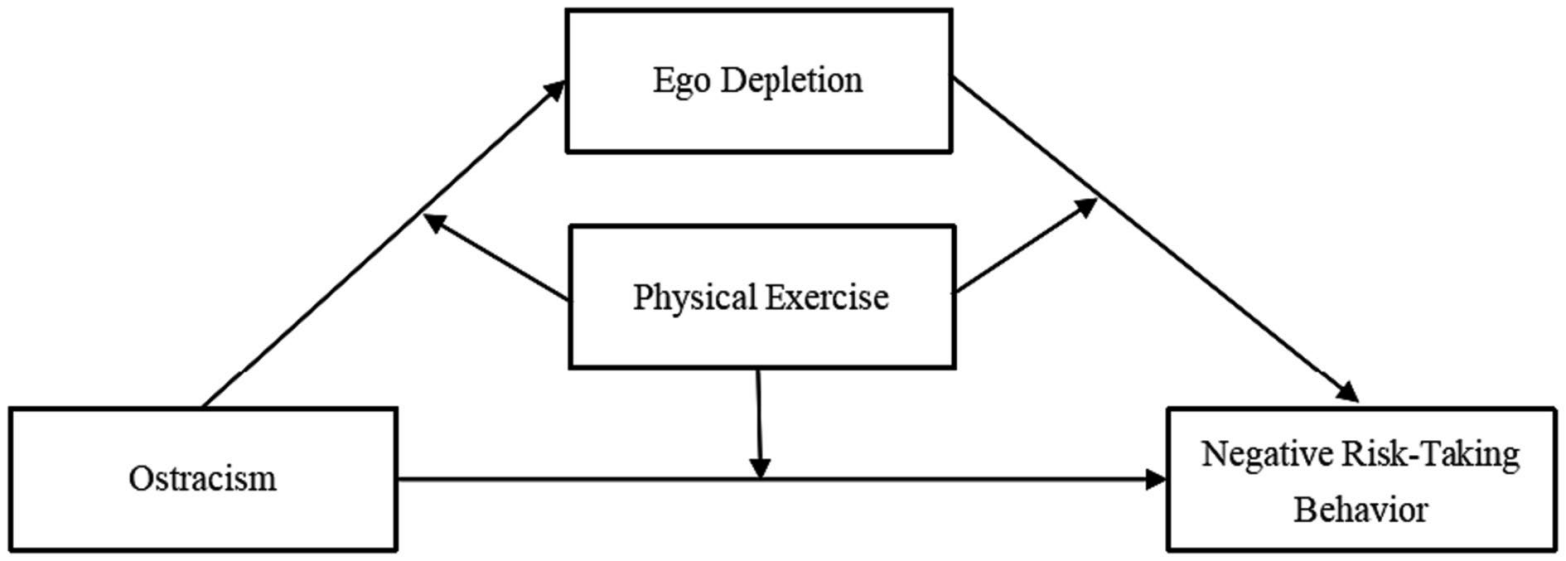
The proposed moderated mediation model.

Many previous studies have examined the independent effects of ostracism or ego depletion on negative risk-taking behaviors among college students, but few studies have combined them and lacked research exploring the protective mechanisms involved. Physical exercise, as a significant factor influencing the mental health and behavior of college students (Tao et al., 2020), it may also have an influence on the relationships between ostracism, ego depletion, and negative risk-taking behavior. Therefore, the present study tested the relationship between ostracism and risk-taking in a sample of college students in China. Using the conceptual frameworks of ego depletion theory and the integrative model of athletic performance, a moderated mediation model (Figure 1) was constructed to test the following hypotheses.

*Hypothesis 1*: Ostracism will be positively related to negative risk-taking behavior.

*Hypothesis 2*: Ego depletion will mediate the relationship between ostracism and negative risk-taking behavior.

*Hypothesis 3*: Physical exercise will moderate the whole effect paths of ostracism on negative risk-taking behavior. Specifically, physical exercise will moderate the association between ostracism and ego depletion, ostracism and negative risk-taking behavior, ego depletion and negative risk-taking behavior.

## 2 Method

### 2.1 Participants and procedure

An online questionnaire was administered to non-physical education majors from four universities in Jingzhou and Wuhan, China, using cluster random sampling, with informed consent from their classroom teachers and students. The primary examiner was a master’s student majoring in psychology. Before any data were collected, the main assessor would provide guidance to the participants and ask them to raise their hands if they had any questions during the answering period, and allow them to stop asking questions at any time without facing any consequences. Meanwhile, subjects were told that this survey was a psychological screening and that it was voluntary and unpaid. Students completed the questionnaires using a mobile application called Sojump (www.sojump.com), and all questionnaires are completed anonymously and results can only be submitted if each question is completed. All participants signed a written informed consent form, and the study was approved by the Ethics Committee of the College of Education and Sports Sciences, Yangtze University.

A total of 1,488 questionnaires was received. Of these, 181 questionnaires were considered invalid and excluded because of the response time (5–25 min) or results (no obvious carelessness in completing the questionnaires); this constituted a validity rate of 87.84%. In the final sample of 1,307 participants, there were 750 (57.38%) men and 557 (42.62%) women. There were 465 (35.58%) freshmen, 445 (34.05%) sophomores, 281 (21.50%) juniors, and 116 (8.87%) seniors.

### 2.2 Measures

#### 2.2.1 Ostracism

Ostracism was measured by the Ostracism Experience Scale for Adolescents (OES-A), which was developed by Gilman et al. (2013) and revised by Niu et al. (2016). This scale consists of 11 items (e.g., “In general, my classmates seem to ignore me.”) that form two dimensions of ignorance and rejection. The scale is scored on a 5-point scale from “1 = strongly disagree” to “5 = strongly agree,” with the final score being the average of the 11 items, with higher scores indicating higher ostracism. In the present study, the Cronbach’s α was 0.85.

#### 2.2.2 Ego depletion

Ego depletion was measured by the Ego Depletion Scale, which was developed by Lanaj et al. (2014) and proved to be widely applicable to Chinese college students (Lanaj et al., 2014; Ding et al., 2020b; Liu et al., 2023). This scale consists of five items (e.g., “I have been feeling low energy lately”) on a 5-point scale, ranging from “1 = not at all” to “5 = completely.” The final score is the average of 5 items, the higher the score, the higher the ego depletion. In the present study, the Cronbach’s α was 0.95.

#### 2.2.3 Physical exercise

Physical exercise was measured by the Physical Activity Rating Scale (PARS-3) (Liang, 1994). This scale consists of three items that measure physical exercise in terms of intensity, time, and frequency. The intensity and frequency of physical exercise, according to the usual situation, are divided into 5 levels and scored from 1 to 5 (light exercise (e.g., walking, radio exercises, etc.)/one time a month or less to intense, continuous and long-lasting exercise (e.g., intense running, martial arts or aerobics exercises, swimming, etc.)/about one times a day), and the duration of physical exercise is scored 0 to 4 (the duration of each physical exercise is 10 min or less to 60 min or more). The level scores for the three items are combined using the formula: Amount of Exercise = Intensity ×Time × Frequency. The higher the score, the higher the level of physical exercise. In the present study, the Cronbach’s α was 0.71.

#### 2.2.4 Negative risk-taking behavior

Negative risk-taking behavior was measured by the Chinese version of the Adolescent Risk-Taking Behavior Scale, which was developed by Gullone et al. (2000) and revised by Zhang et al. (2011). The measure has four parts, namely stimulus seeking, recklessness, rebellion, and antisociality. The three remaining parts are thought to represent negative risk-taking behaviors, whereas stimulus seeking activities are thought to be positive or socially acceptable (Moore et al., 2004). Following the practice of previous studies (Liu et al., 2019; Jia et al., 2021), only the last three negative dimensions, with a total of 12 items (e.g., “making fun of or bullying others”), were used as indicators of negative risk-taking behavior. Each item is scored on a 5-point scale, ranging from “0 = never” to “4 = always.” The final score is the average of 12 items for recklessness, rebelliousness, and antisociality, with higher scores indicating higher levels of negative risk-taking behavior. In the present study, the Cronbach’s α was 0.94.

### 2.3 Data analysis

First, SPSS 26.0 was used to test common method bias and collinearity and to generate descriptive statistics and Pearson correlations among study variables. Second, all variable scores were standardized and the hypotheses were tested using the PROCESS 3.4 macro in SPSS 26.0 (Hayes, 2013). Hypothesis 1 and 2 (regarding mediation) were tested by Model 4 in the macro, and Hypothesis 3 (regarding moderated mediation) was tested in Model 59 in the macro. Last, the two models were tested using a bias-corrected percentile Bootstrap method with 95% confidence interval. An effect is considered significant when the confidence interval does not include 0. Gender and grade were controlled for in in tests of the hypotheses because previous studies found them to be closely related to the main variables of the current study (García et al., 2014; Ding et al., 2020a; Sun et al., 2021).

## 3 Results

### 3.1 Common method bias and collinearity

All data were collected from participants’ self-reports, raising the concern of common method bias. An exploratory factor analysis of all items from all of the self-report measures (Ostracism Experience Scale for Adolescents, Ego Depletion Scale, Physical Activity Rating Scale, and Negative Risk-taking Behavior Scale) identified five factors with characteristic roots greater than 1. The first factor explained 36.50% of the variance in this set of scores. According to Harmon’s single factor test, this value is lower than the cutoff of 40%, suggesting that there was no substantial common method variance.

### 3.2 Collinearity test

Because the analyses used linear regression and the main study variables were significantly inter-correlated, two tests of collinearity were conducted. Tolerance was greater than 0.1 (0.84–1.00) and the variance inflation factor (VIF) was less than 3 (1.04–1.19), indicating no serious multicollinearity.

### 3.3 Preliminary analyses

The descriptive statistics and correlations are shown in Table 1. Ostracism was positively correlated with ego depletion and negative risk-taking behavior (*r* = 0.25, 0.27, *p* < 0.001), ego depletion was negatively correlated with negative risk-taking behavior (*r* = 0.37, *p* < 0.001), and physical exercise was negatively correlated with ego depletion and negative risk-taking behavior (*r* = −0.34, −0.47, *p* < 0.001).

**Table 1:** Means, standard deviations, and correlations among the main study variables.

	*M*	SD	1	2	3	4	5	6
1. Gender	1.43	0.50	1					
2. Year in college	2.04	0.96	−0.01	1				
3. Ostracism	2.40	0.70	−0.10^***^	0.04	1			
4. Ego depletion	2.36	1.07	0.12^***^	0.01	0.25^***^	1		
5. Physical exercise	26.87	20.86	−0.09^**^	−0.04	−0.26^***^	−0.34^***^	1	
6. Negative risk-taking behavior	0.47	0.66	−0.12^***^	0.01	0.27^***^	0.37^***^	−0.47^***^	1

*N* = 1,574; **p < 0.01, ***p < 0.001; for gender, “1” = “male” and “2” = “female”.

### 3.4 Testing for mediation effect

**Figure 2:**
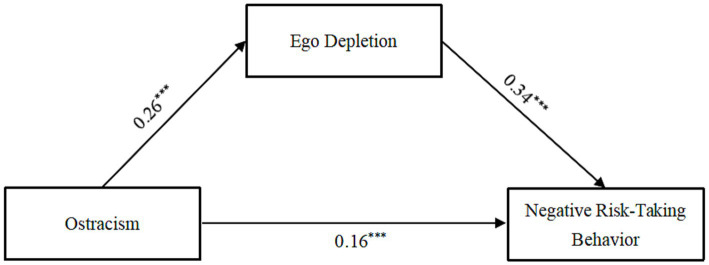
Mediation analysis. ^***^p < 0.001.

Model 4 in the SPSS PROCESS 3.4 macro (Hayes, 2013) was used to analyze the mediation effect. In this study, it assumes that the independent variable (ostracism) will have an effect on the dependent variable (negative risk-taking behavior) through the mediating variable (ego depletion) and thus the dependent variable. The main variables in the data were standardized and after controlling for gender and grade, the results are shown in Table 2 and Figure 2. In support of Hypothesis 1, ostracism was associated with negative risk-taking behavior (*β* = 0.25, *p* < 0.001). In partial support of Hypothesis 2, this association was lower, but remained significant, after including ego depletion as a mediator (*β* = 0.16, *p* < 0.001). Ostracism was positively associated with ego depletion (*β* = 0.26, *p* < 0.001); ego depletion was positively associated with negative risk-taking behavior (*β* = 0.34, *p* < 0.001). In addition, the bootstrap 95% confidence interval did not include 0 either for the direct effect of ostracism on negative risk-taking behavior (0.11, 0.21), or for the indirect effect via ego depletion (0.07, 0.12). Thus, ostracism not only directly predicted negative risk-taking behavior, but also indirectly predicted negative risk-taking behavior through ego depletion, with ego depletion playing a partially mediating role. The direct and indirect effects contributed 64.00 and 36.00% to the total effect, respectively (Table 3).

**Table 2:** Regression analysis of the mediating effect of ego depletion.

Regression equation		Overall fitting index			Regression coefficient	
Dependent variable	Independent variable(s)	*R*	*R* ^2^	*F*	*β*	*t*
Negative risk-taking behavior		0.28	0.08	37.02^***^		
	Gender				−0.18	−3.33^***^
	Year in college				0.00	−0.001
	Ostracism				0.25	9.60^***^
Ego depletion		0.29	0.08	40.18^***^		
	Gender				0.30	5.39^***^
	Year in college				−0.002	−0.07
	Ostracism				0.26	10.06^***^
Negative risk-taking behavior		0.44	0.19	76.23^***^		
	Gender				−0.28	−5.49^***^
	Year in college				0.001	0.02
	Ostracism				0.16	6.27^***^
	Ego depletion				0.34	13.37^***^

*N* = 1,574; ***p < 0.001; for gender, “1” = “male” and “2” = “female”.

**Table 3:** Mediation effect test.

Pathway	Effect	Boot SE	Boot LLCI	Boot ULCI	Proportion of effect
Total effect	0.25	0.03	0.20	0.30	
Direct effect	0.16	0.03	0.11	0.21	64.00%
Indirect effect	0.09	0.01	0.07	0.12	36.00%

### 3.5 Moderated mediation effect analysis

**Figure 3:**
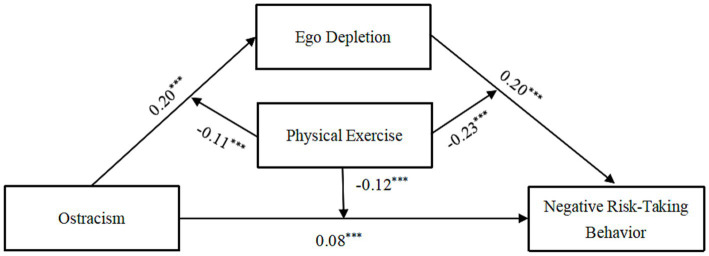
Moderated mediation analysis. ^***^p < 0.001.

Hypothesis 3 proposed a moderated mediation model. This moderated mediation model was tested using Model 59 in SPSS PROCESS 3.4 (Hayes, 2013), which assumes that the direct path and indirect path are all moderated by the physical exercise, which is consistent with the assumptions of this study. The main variables in the data were standardized and incorporating physical exercise as a moderating variable based on the mediation model, the results are presented in Figure 3 and Table 4. The interaction between ostracism and physical exercise was a significant predictor of both ego depletion (*β* = −0.11, *p* < 0.001) and negative risk-taking behavior (*β* = −0.12, *p* < 0.001); the product of ego depletion and physical exercise was significantly related to negative risk-taking behavior (*β* = −0.23, *p* < 0.001).

**Table 4:** Regression analysis of the moderating effect of physical exercise.

Regression equation		Overall fitting index			Regression coefficient	
Dependent variable	Independent variable(s)	*R*	*R* ^2^	*F*	*β*	*t*
Ego depletion		0.42	0.17	54.52^***^		
	Gender				0.26	4.86^***^
	Year in college				−0.01	−0.25
	Ostracism				0.20	7.79^***^
	Physical exercise				−0.28	−10.86^***^
	Ostracism × physical exercise				−0.11	−5.06^***^
Negative risk-taking behavior		0.63	0.39	120.36^***^		
	Gender				−0.26	−5.79^***^
	Year in college				−0.01	−0.38
	Ostracism				0.08	3.47^***^
	Ego depletion				0.20	8.47^***^
	Physical exercise				−0.43	−18.63^***^
	Ostracism × physical exercise				−0.12	−6.38^***^
	Ego depletion × physical exercise				−0.23	−10.49^***^

*N* = 1,574; ***p < 0.001; for gender, “1” = “male” and “2” = “female”.

**Figure 4:**
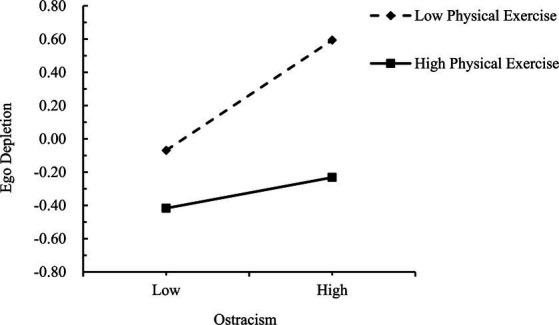
Moderating effect of physical exercise on the relationship between ostracism and ego depletion.

**Figure 5:**
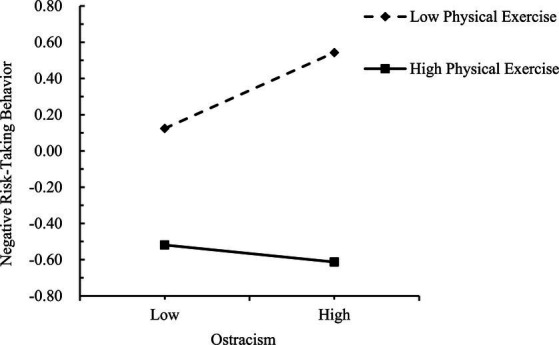
Moderating effect of physical exercise on the relationship between ostracism and negative risk-taking behavior.

**Figure 6:**
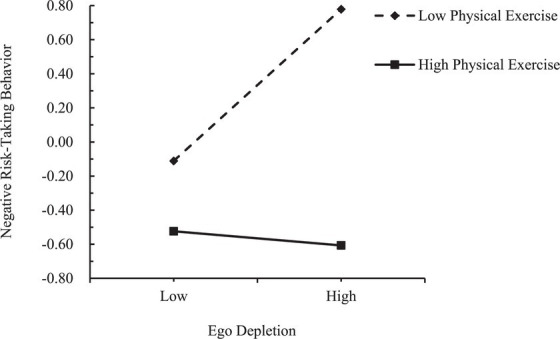
Moderating effect of physical exercise on the relationship between ego depletion and negative risk-taking behavior.

Simple slopes tests were used to interpret these significant interactions. Physical exercise scores were dichotomized as low (*M* − 1 *SD*) or high (*M* + 1 *SD*). As shown in Figure 4, the predictive effect of ostracism on ego depletion was significantly higher for individuals with low physical exercise (*β* = 0.32, *t* = 8.76, *p* < 0.001) than for those with high physical exercise (*β* = 0.09, *t* = 2.72, *p* < 0.01), indicating that the effect of ostracism on ego depletion was weaker as physical exercise increased. As shown in Figures 5, 6, in the low physical exercise group there were significant effects of ostracism (*β* = 0.20, *t* = 6.34, *p* < 0.001) and ego depletion (*β* = 0.43, *t* = 13.76, *p* < 0.001) on negative risk-taking behavior. However, in the high physical exercise group, ostracism (*β* = −0.05, *t* = −1.65 *p* > 0.05) and ego depletion (*β* = −0.04, *t* = −1.22, *p* > 0.05) were not significantly associated with negative risk-taking behavior.

## 4 Discussion

### 4.1 Ostracism and negative risk-taking behavior

The findings showed that ostracism was associated with more negative risk-taking, which supported Hypothesis 1 and was consistent with previous research (Meng, 2020). Ostracism appears to be a significant risk factor for negative risk-taking behavior among Chinese college students, which is consistent with the limited-resource theory (Baumeister, 2003). Ostracism can have negative effects on an individual’s self-control resources and self-regulation ability. These have a close relationship with people’s decision-making behavior in response to complex risk situations, it is a significant predictor of negative risk-taking behavior among college students (Xu et al., 2015). In addition, ostracism as a stressful event can cause adolescents to develop negative emotions (anger, frustration and sadness) or to undermine basic psychological needs in order to alleviate emotions and maintain psychological equilibrium through stimulating, rebellious, and antisocial risk-taking behaviors (Williams, 2009; DeBono and Muraven, 2014; He et al., 2021). Examples include smoking, drinking, drug use, fighting and truancy. Therefore, how to prevent and reduce the occurrence of ostracism is a key concern for families and schools. Reducing ostracism and promoting harmonious interpersonal relationships can be an effective way to prevent college students from engaging in negative risk-taking behaviors. Simultaneously, some studies have shown that cumulative ecological risk affects college students’ risky behaviors, and the more stressful the external environment is, the higher the incidence of risky behaviors (Dou et al., 2020a; Hu et al., 2023). This suggests that apart from ostracism, attention should also be paid to reducing other ecological risk factors in the developmental environment of college students (e.g., family support, faculty-student relationships, and school connections) to control the occurrence of their negative risk-taking behavior.

### 4.2 The mediating role of ego depletion

Consistent with Hypothesis 2, ego depletion mediated the relationship between ostracism and negative risk-taking behavior, which is similar to a previous study (Dewall et al., 2012; Buelow and Wirth, 2017). That is, once college students consume excessive self-control resources to defend themselves against external pressures from or after ostracism, they are prone to ego depletion, which leads to negative risk-taking behavior. This suggests that we should think about how to go about enhancing college students’ self-control resources and preventing ego depletion. The results likewise provide empirical support for the limited resources theory. The theory suggests that external stressful events (ostracism) can cause stress to individuals. The processes of coping with stress (e.g., suppressing emotions, monitoring stimuli, denial, etc.) all require a person to change or stop current thoughts, emotions, or behaviors. This is a prerequisite for exhausting self-control resources. Adapting to stress can deplete self-control resources even when the stress itself is over. The consequence of depletion is a loss of self-control and difficulty in suppressing the desire and urge to engage in risk-taking behavior. This demonstrates that depletion of self-control resources is an important potential mechanism by which ostracism influences negative risk-taking behavior among college students. This finding also holds true for online interpersonal relationships in the information age, where it has been demonstrated that online social exclusion depletes an individual’s limited resources of self-control and inability to regulate perceptions, emotions, and behaviors about themselves, thus increasing the risk of externalizing problematic behaviors (Huang and Xin, 2023).

In our study, ostracized college students were more prone to ego depletion whether they were coping with distress by repressing or regulating negative emotions or maintaining psychological balance and gaining a sense of being. This is because these demands and efforts depleted their self-control resources (Baumeister, 2003; Huang and Xin, 2023). This finding supports previous research that excluded college students experience distressing and negative emotions, and self-regulation requires mobilization of resources in the self-control system, and that a lack of resources in subsequent cognitive processes increases the likelihood of ego depletion (Baumeister et al., 2005). College students in ego depletion have weakened attention and willpower, and their daily tolerance and judgment are reduced, with which behavioral inhibition is weakened, thus they may engage in potentially destructive behaviors, such as increased alcohol consumption and excessive alcohol consumption, drug use, aggression, or impulsive spending (Bishop, 2017; Dou et al., 2020b). This also supports the ego depletion theory, which states that an individual’s ability to regulate behavior decreases in a state of ego depletion, further affecting the individual’s behavior (Baumeister, 2014). Therefore, ego depletion is an important bridge between ostracism and negative risk-taking behavior. The likelihood of negative risk-taking behavior can be reduced by reducing college students’ ego depletion.

### 4.3 The moderating role of physical exercise

In the current study, physical exercise level (intensity, frequency, duration) attenuated the effects of ostracism on negative risk-taking behavior. This finding is consistent with Hypothesis 3, and partially consistent with the results of a related study (Schoendube et al., 2017). Physical exercise also weakened the influence of ego depletion as a mediator of the association between ostracism and risk-taking. The results are consistent with the integrative model of athletic performance (Huang et al., 2023). According to this model, physical exercise, as an activity beneficial to physical and mental health, can enhance positive emotional experiences and self-control by mitigating the influence of unfavorable external factors. In the case of the current study, this external factor was the experience of ostracism.

The self-control energy model also supports the results，which maintains that physical exercise can help individuals regulate their self-control, reduce the depletion of their self-control resources, and free themselves from repression and impulsivity (Wang et al., 2022). Earlier studies have also demonstrated that exercise can increase the amount and endurance of self-control, and motor exercise immediately prior to cognitive skill training can aid in the rapid recovery of cognitive control resources during subsequent skill completion (Ludyga et al., 2018). These results, and our own, suggest that reasonable physical exercise can enhance the self-control resource reserves and reduce the risk of entering into ego depletion after the experience of ostracism.

Similarly, studies have pointed out that physical exercise can help college students cope with external risk factors (ostracism) to inhibit risk-taking behavior. This because physical exercise can enhance cognitive abilities, social interaction skills, and emotion management skills (Culpepper and Killion, 2017; Tao et al., 2023). They can effectively distract themselves from stressful stimuli, counteract negative emotions, and improve negative cognition to Khoury-Kassabri and Schneider (2018) and Watson et al. (2017). In addition, a study has shown that part of the risk-taking behavior can also be released through the mental and physical energy of physical exercise (physical fitness and impulsive desire, rather than the self-control resource) (Park et al., 2021). In fact, this is also a process of controlling the self and detaching from depletion, which is beneficial for the purpose of preventing and controlling risk-taking behavior. This suggests that physical exercise can help college students cope with negative risk-taking behavior associated with ostracism and ego depletion and is a protective factor for physical and mental health. Nevertheless, the phenomenon of lack of exercise among college students in China is widespread, for example, a recent large-sample cross-sectional study revealed that as high as 76.0% of college students lacked exercise (Hu et al., 2023). Therefore, it is very necessary to encourage college students to participate in sports and scientifically guide them to exercise reasonably.

However, high-level physical exercise did not moderate the relationships among ostracism, ego depletion, and negative risk-taking behavior. This could be because medium physical exercise is a value threshold that has been recognized by national and international studies (Bull et al., 2020). Previous studies have shown that moderate and low-level physical exercise have a more pronounced positive effect on individual psychology and behavior, and exercise intensity has an inverted U-shaped relationship with cognitive function (Wang et al., 2016). Specifically, high-level physical exercise may lead to transient cognitive impairment, an inability to regulate problematic behaviors caused by adverse factors, and possibly even negative outcomes (drinking, fighting, etc.) (Wang and Gathercole, 2013; Peng, 2022). In addition, there are certain commonalities among participation in sports, physical exercise, and risk-taking behavior. Athleticism, especially when competitive, commonly involves aggression (Black et al., 2013). College students may not benefit from the positive effects of physical exercise if they are in this environment for a prolonged period. Therefore, proper physical exercise is a beneficial factor, but we should try to avoid high-intensity, high-frequency, and high-duration exercise.

### 4.4 Limitations and ideas

This study is not without limitations. First, the effect of ostracism on negative risk-taking behavior may be related to the type and duration of ostracism, and these characteristics were not assessed (I am making this up) “Ostracism by residents in a college dormitory may be especially distressing to students.” Second, the results of this study are limited by the cross-sectional design, which may compromise the establishment of a causal relationship. In China, negative risk-taking behavior is not recognized by the general public; therefore, it is possible that negative risk-taking behavior can lead to the ostracism of individuals. Longitudinal and experimental studies will be helpful in further delineating the association between ostracism and risk-taking behavior. For example, an experiment that ostracism was manipulated through the Cyberball paradigm, and risk-taking behavior was measured using Balloon Analog Risk Task, which confirmed the positive effect of ostracism on risk-taking behavior. Third, the study did not measure the type and duration of ostracism, and used a composite measure of risk-taking that could not identify specific risky behaviors associated with ostracism.

## 5 Conclusion

In this study, a moderated mediation model was tested in order to explore the relationship between ostracism and negative risk-taking behaviors of Chinese college students and its internal mechanisms. The study has demonstrated that ostracism was positively associated with Chinese college students’ negative risk-taking behavior. Moreover, ego depletion mediated the relationship between ostracism and negative risk-taking behavior. Simultaneously, low and moderate physical exercise was a protective factor that mitigated these direct and indirect effects.

The results of this study have implications for current models’ conceptualizations of the effects of ostracism. Tests of the moderated mediation model provided evidence of how (the mediating role of ego depletion) and when (the moderating role of physical exercise) ostracism influences Chinese college students’ negative risk-taking behavior. Baumeister’s limited resource theory provides a good framework for the mediating mechanism in this study, which is also enriched with evidence related to the kind of external stressful environments that ego depletion can be influenced by. Additionally, we combined physical exercise with ego depletion mechanisms, which greatly enriches limited resource theory and theories about physical exercise as an individual protective factor.

The results also have some practical implications for fostering the wellbeing of college students. Ostracism is a direct risk factor for ego depletion and negative risk-taking behavior. College students could be educated on the effects of ostracism and alternatives to ostracizing group members. The prevention of ego depletion could be included in any self-care initiatives or programs designed to reduce risk-taking behaviors such as alcohol abuse. Students can also be encouraged to engage in physical exercise and sports as a way to strengthen self-regulation. One example is the “trail running app” adopted by some universities in Hubei Province in China. This app uses a credit system to reward daily exercise.

## Data availability statement

The datasets presented in this article are not readily available because the datasets generated during and/or analyzed during the current study are available from the corresponding author on reasonable request. Requests to access the datasets should be directed to HZ, zouhongyu2016@163.com.

## Ethics statement

The studies involving humans were approved by the ethics committee of Yangtze university. The studies were conducted in accordance with the local legislation and institutional requirements. The participants provided their written informed consent to participate in this study.

## Author contributions

FC: Writing – original draft. JW: Writing – original draft. HG: Writing – review & editing. YZ: Writing – review & editing. ZL: Writing – review & editing. HZ: Writing – review & editing.
